# The Trend of Healthcare Needs among Elders and Its Association with Healthcare Access and Quality in Low-Income Countries: An Exploration of the Global Burden of Disease Study 2019

**DOI:** 10.3390/healthcare11111631

**Published:** 2023-06-02

**Authors:** Joshua Kirabo Sempungu, Minjae Choi, Eun Hae Lee, Yo Han Lee

**Affiliations:** 1 Program in Public Health, Graduate School, Korea University, 73 Goryeodae-ro, Seongbuk-gu, Seoul 02841, Republic of Korea; sjkirabo@korea.ac.kr (J.K.S.);; 2 Department of Preventive Medicine, Korea University College of Medicine, 73 Goryeodae-ro, Seongbuk-gu, Seoul 02841, Republic of Korea; 3 Institute for Future Public Health, Graduate School of Public Health, Korea University, 73 Goryeodae-ro, Seongbuk-gu, Seoul 02841, Republic of Korea

**Keywords:** life expectancy, ageing, age-friendly healthcare system, low-income countries, global burden of disease

## Abstract

To investigate the trend of healthcare needs among elders in low-income countries (LICs) and how changes in healthcare access and quality (HAQ) have correlated with these changes from 1990 to 2019, this study used estimates from the global burden of disease (GBD) 2019 study, including prevalence, years of life lost (YLLs), years lived with disability (YLDs), life expectancy (LE), health-adjusted life expectancy (HALE) and the HAQ index for years 1990 and 2019. We found increases in numbers of YLLs, YLDs, and prevalent cases due to NCDs, and the rate of increase was higher for all indicators of non-communicable diseases (NCDs) when compared with communicable, maternal, neonatal and nutritional diseases among elders. We also observed increases in LE and HALE among all countries. However, this was also challenged by increases in unhealthy life years (ULYs) and their constant percentage of LE. The HAQ index of LICs was also found to be low, although it had increased during the period. A reduction in the burden of acute diseases explains the increase in LE, but increases in ULYs and the NCD burden were also observed. LICs need to improve their HAQ to counter the growing threat of longer but less healthy lives.

## 1. Introduction

Aging is one of the biggest concerns of our time around the globe. This is simply because many developed nations have experienced population transitions into aged societies, and the World Health Organization (WHO) projects that by 2050, one in every five people will be over 60 years old [1]. As the focus on aging continues to be in high-income countries (HICs), low-income countries (LICs) (countries with a gross national income per capita less than or equal to 1085 US dollars in 2021) [2] usually feel unbothered, but indeed the populations in these countries too are aging. Nonetheless, numerous studies conducted in aging societies indicate the need for shifts in healthcare provision and policies to match the increased need for healthcare that comes with increases in aged populations [3,4,5].

The population of elderly people was projected to be around 40 million people in low-income countries in 2022, but this is expected to increase to 100 million people in 2050 [6]. This increase in the number of older people is easily attributable to longer life expectancy, which is a result of the successful minimization of premature mortality. In their study, which included 54 low-income countries, Hauck et al. noted that life expectancy increases were associated with a reduction in the human immunodeficiency virus prevalence among children, advances in gender equality, agricultural production and political stability, among other reasons [7]. These advances have led the life expectancy of low-income countries to increase from 50 years in 1990 to 63 years in 2020 [8].

At the same time, the burden of disease among the elderly is also high. People aged 55 years or more accounted for 39.5% of the global total number of disability-adjusted life years (DALYs) in 2019, and 15.2% of the DALYs in low social development index countries [9]. Among contributors to the DALYs in low-income countries, it was noted that in 2010, cardiovascular diseases contributed the most, followed by cancer; however, low-income countries still suffered from a “prominent contribution” of infectious and parasitic diseases [10].

As the population ages, healthcare needs have also risen tremendously. Outside low-income countries, highly developed nations have taken the stand and implemented policies to counter the increasing burden of health care needs, and their interventions include the setup of aging population cohort studies, social security funds and the boost of elderly care in national health insurance policies [3,5,11]. Nevertheless, even with these interventions, there remains a gap in achieving a long, healthy life in many countries. 

Studies have tried to explain the issue of lengthy living in comparison with healthy living. Fries in 1980 found that as life expectancy increased, there was a reduction in mortality due to acute disease, but measures of chronic diseases increased. In addition, in coining the notion of “compression of morbidity”, the issue of longer life and later onset of both death and disability were notable factors [12]. Rachel et al. highlighted three scenarios to explain potential outcomes of increased life expectancy: in their series, expansion of morbidity was a scenario in which increasing life expectancy would lead to the increased time spent in ill health; then, similar to Fries, they argued that compression of morbidity would cause a later onset of disease, thus a smaller proportion of life lived would be spent in illness [13]. 

Although the delayed onset of disease may be desirable, most aging societies are developed nations with functional healthcare systems. Low-income countries, on the other hand, still struggle with healthcare service provision to their populations, where over 50% of healthcare financing has been estimated to be out of pocket and there is low insurance coverage for the populations [14]. Many of these LICs still have low employment rates and poor social security systems, both of which have been noted as threats to the aging population, and as Ziegenhain et al. pointed out, these countries are not ready for aging [15].

Our study aims to show the trend of the burden of healthcare needs, including prevalence, years lived with disability, years of life lost and unhealthy life years, and the trend of life expectancy and health-adjusted life expectancy in contrast with the healthcare access and quality index and their correlation among elders in low-income countries.

## 2. Materials and Methods

### 2.1. Data Sources

The data used in this study was extracted from the Institute of Health Metrics and Evaluation’s (IHME) Global Burden of Disease (GBD) 2019 database. The GBD 2019 study provides data that is used to measure countries’ health challenges and how they vary over time and among countries, regions and subregions, among other classifications. The GBD 2019 study included the categorization of 369 diseases and injuries and attributed burden to 87 risk factors from 204 countries and territories categorized by sex and age groups for indicators including incidence, prevalence, deaths, DALYs, years of life lost (YLL), years lived with disability (YLD), life expectancy and health-adjusted life expectancy (HALE) for the period between 1990 and 2019. Previous studies have described the methodological construction of the GBD 2019 [16]. The GBD 2019 study also categorized diseases and injuries into four-level strata, with each level consisting of mutually exclusive causes. Level 1 causes are grouped into three groups: communicable, maternal, neonatal and nutritional diseases (CMNNDs); non-communicable diseases (NCDs); and injuries. Levels 2, 3 and 4 comprise 22, 174 and 301 causes, respectively [17]. The GBD has been noted to have used all available data sources and, having assessed the quality of each source to eliminate biases, used sound statistical modeling tools and methods to generate 95% uncertainty intervals (UIs) indicating the 2.5th and 97.5th percentiles in the distribution around the mean estimates [17]. Our study adhered to the guidelines for accurate and transparent health estimate reporting (GATHER) [16,17].

### 2.2. Country Inclusion Criteria

The GBD study does not categorize LICs, so we used the World Bank categorization, which includes 28 nations, including Afghanistan, Burkina Faso, Burundi, Central African Republic (CAR), Chad, Democratic Republic of the Congo (DRC), Eritrea, Ethiopia, Gambia, Guinea, Guinea-Bissau, Liberia, Madagascar, Malawi, Mali, Mozambique, Niger, Democratic People’s Republic of Korea (North Korea), Rwanda, Sierra Leone, Somalia, South Sudan, Sudan, Syrian Arab Republic (SAR), Togo, Uganda, Yemen and Zambia. The World Bank’s 2023 classification of LICs considers countries with a gross national income (GNI) per capita lesser than or equal to 1085 US dollars in 2021, and all 28 countries included in that classification are included in this study [2]. The classification of lower middle-income countries includes countries with a GNI per capita between 1086 and 4255 US dollars, upper middle-income countries as those with a GNI per capita between 4256 and 13,205 US dollars, and high-income countries as those with a GNI per capita of 13,205 US dollars or more. Further information about methods used by the World Bank to determine income classification has been discussed exhaustively in a previous article by Fantom and Serajuddin in 2016 [18].

### 2.3. Data Processing

This study included estimates of YLDs, YLLs and prevalence at ages 65–74. YLDs are calculated by multiplying prevalence estimates of a defined disease by a corresponding disability weight; YLLs are estimated by multiplying the number of deaths by the remaining life expectancy at the age of death. Prevalence has been noted to be aggregated in estimation at the level of individuals who may have more than one sequela or disease in this study [17]. Health-adjusted life expectancy (HALE) and life expectancy included in this study are GBD estimates of life expectancy at birth and HALE at birth for all countries. Unhealthy life years (ULYs) were calculated by subtracting HALE from life expectancy for every year (ULY = life expectancy – HALE). The proportion of ULYs on life expectancy was computed as a percentage ((life expectancy – HALE)/life expectancy).

To estimate the performance of healthcare systems in the LICs, we used the healthcare access and quality (HAQ) index, a measure that has been found to have stronger convergency validity when compared to other health-system indicators. The methods and analytic framework of the HAQ estimation have been published elsewhere [19]. The HAQ was calculated for three select age groups: young (0–14 years), working (15–64 years) and post-working (65–74 years). This study used the HAQ index of the post-working age group. 

All data used in this study to estimate YLLs, YLDs, prevalence and HAQ are downloadable at the global health data exchange (http://ghdx.healthdata.org/gbd-results-tool (accessed on 2 February 2023)).

### 2.4. Measures

To estimate the average annual percentage change (AAPC), we utilized the joinpoint regression program version 4.9.1.0. The Joinpoint regression program is a trend analysis software developed by the US National Cancer Institute for the analysis of data from the surveillance epidemiology and results program [20,21]. The program fits a series of joined straight lines on a logarithmic scale, and segments are joined at “joinpoints”. The joinpoints range from 0 to 5. The slope of each line segment of the best-fitting model was expressed as the annual percentage change (APC), and AAPC was expressed as a summary measure over a fixed interval. The joinpoint methodology for the estimation of APCs and AAPCs has been documented in previous publications [22,23].

## 3. Results

### 3.1. The Trend of Communicable, Maternal, Neonatal and Nutritional Diseases

Table 1 indicates that most countries had increases in the number of YLDs, with the highest AAPC being that of Somalia at 3.9 (3.9–4) due to an increase from 3111.32 (2156.27–4273.46) YLDs in 1990 to 9542.08 (6676.76–13,138.48) in 2019. Other countries with high AAPCs included the Syrian Arab Republic (3.2 (3.1–3.4)), North Korea (2.6 (2.4–2.8)) and Gambia (2.5 (2.4–2.7)). On the other hand, some countries recorded decreases in the number of YLDs; these included Burkina Faso, where the highest reduction rate was registered, bringing the number of YLDs from 12,379.66 (8719.11–16,848.82) in 1990 to 7993.78 (5705.16–10,695.89) in 2019 with an AAPC of −1.4 (−1.7–1.1). Other countries with negative AAPCs included Mali’s −0.8 (−1–0.7), Guinea’s −0.7 (−0.8–0.6) and Guinea-Bissau’s −0.4 (−0.5–0.3). 

All countries had increases in prevalence due to CMNNDs, but once again Somalia had the highest rate of increase with an AAPC of 4.1 (4.1–4.2), and prevalent cases increased from 92,648.78 (90,464.77–94,574.35) in 1990 to 296,987.07 (288,447.66–306,067.20) in 2019. Another significant rate of increase included Eritrea’s 3.3 (3–3.5), Togo’s 3.3 (3–3.6) and Niger’s 3.1 (2.2–4) AAPCs between 1990 and 2019.

YLLs were found to have decreased in 10 of the 28 studied countries. Ethiopia had an AAPC of −2.3 (−2.5–2.1) with a reduction of YLLs from 711,309.82 (604,381.4–822,846.88) in 1990 to 359,723.85 (307,975.63–419,789.92) in 2019. This AAPC was only followed by Rwanda’s −1.8 (−2.2–1.3) and Afghanistan’s −1.3 (−1.5–1.1). On the contrary, Somalia still had the highest increase in YLLs, with an AAPC of 2.9 (2.5–3.3), over double the number of YLLs compared to 1990. 

### 3.2. The Trend of Non-Communicable Diseases

Table 2 illustrates that all healthcare needs indicators have increased in all LICs during the past 30 years. YLDs had increased in Somalia with an AAPC of 4.3 (4.3–4.4), representing an increase from 17,203.1 (13,106.99–21,954.16) in 1990 to 58,493.42 (44,511.47–75,094.27) in 2019, followed by AAPC increases in Togo (3.9 (3.6–4.3)), Niger (3.8 (2.9–4.6)), Eritrea (3.6 (3.4–3.9)) and Yemen (3.5 (3.4–3.6)). Similar increases in YLDs due to NCDs were recorded, with Somalia’s AAPC of 4.2 (4.2–4.3) remaining the highest rate of increase, followed by Togo (3.8 (3.7–3.9)), Eritrea (3.6 (3.3–3.8)), Niger (3.5 (2.6–4.4)) and Yemen (3.3 (3.3–3.4)). 

YLLs also increased in all countries, and AAPCs were highest in Somalia with 3.7 (3.6–3.8), followed by Gambia (3.6 (2.6–4.5), Togo (3.4 (2.8–4.1)), Eritrea (3.3 (2.7–3.8)) and Niger (3 (2.3–3.7)).

Overall, the AAPCs of NCDs were higher than those of CMNNDs in all the countries on all indicators. Suggesting a higher rate of increase in the burden of NCDs compared to that of CMNNDs.

### 3.3. Trend of Life Expectancy, Health-Adjusted Life Expectancy and Unhealthy Life Years

**Figure 1:**
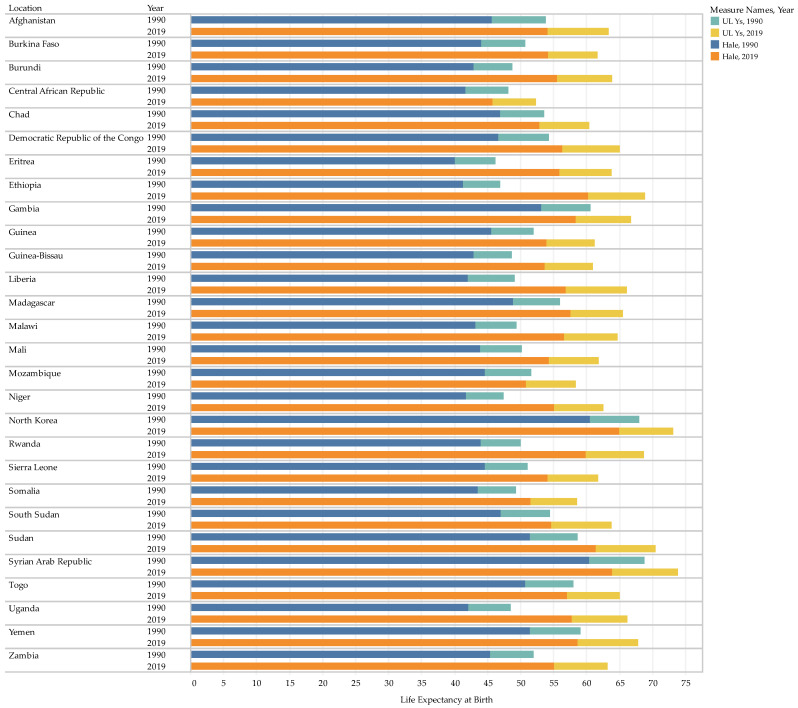
The Trend of Life Expectancy, HALE and Projected Unhealthy Lived Years at Birth between 1990 and 2019.

Life expectancy, HALE and ULYs increased in all countries during the study period. Figure 1 shows that although 10 countries had a life expectancy at birth below 50 years in 1990, all countries had raised their life expectancy, with only three countries having it below 60 in 2019. North Korea and Syria had the highest Life expectancies in the study period, with 67.97 and 68.79 in 1990, then 73.15 and 73.88 in 2019, respectively. The highest rate of increase was recorded in Ethiopia with an AAPC of 1.3 and Eritrea with 1.2, followed by Uganda and Rwanda, both with a 1.1 AAPC for life expectancy. HALE had a similar trend as life expectancy increased at seemingly similar rates throughout all the countries. 

Table 3 shows that all countries also experienced increases in ULYs, although the proportion of these on life expectancy remained similar throughout the study. The proportion of ULYs on life expectancy stayed between 11% and 15% in all 30 years, indicating no change in the proportion of HALEs on life expectancy. 

### 3.4. The Trend of Healthcare Access and Quality

Table 4 shows that although the HAQ index has increased in all 28 studied countries. Significant increases in Ethiopia from 18.22 (13.41–24.68) in 1990 to 23.13 (18.75–28.15) in 2019, implying a 49% increase in the index. Rwanda (43%), Afghanistan (25%) and the Syrian Arab Republic (SAR) (24%) all followed in percentage change. Although both Ethiopia and SAR reported absolute changes of 14.19 and 14.5, respectively. The highest HAQ index remains in the same countries throughout the period, with SAR, North Korea, Sudan and Yemen leading in terms of index scores.

### 3.5. Association between the HAQ and Health Care Needs

**Figure 2:**
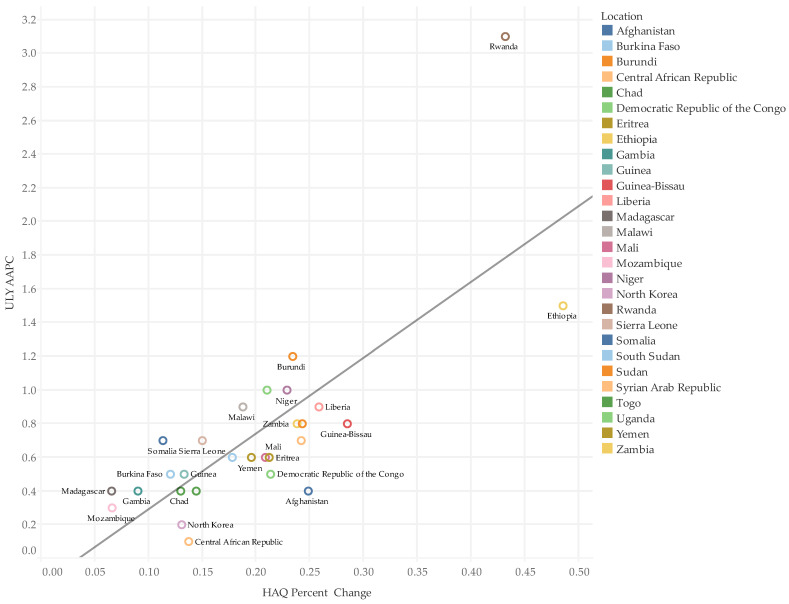
Correlation Between ULY AAPCs and Percentage Change in HAQ. HAQ percentage change = (HAQ in 1990/HAQ in 2019)/HAQ in 1990; ULY AAPC = annual average percentage change of unhealthy life years per country from 1990 to 2019. Trend line: R-squared = 0.59, *p*-value ≤ 0.0001.

**Figure 3:**
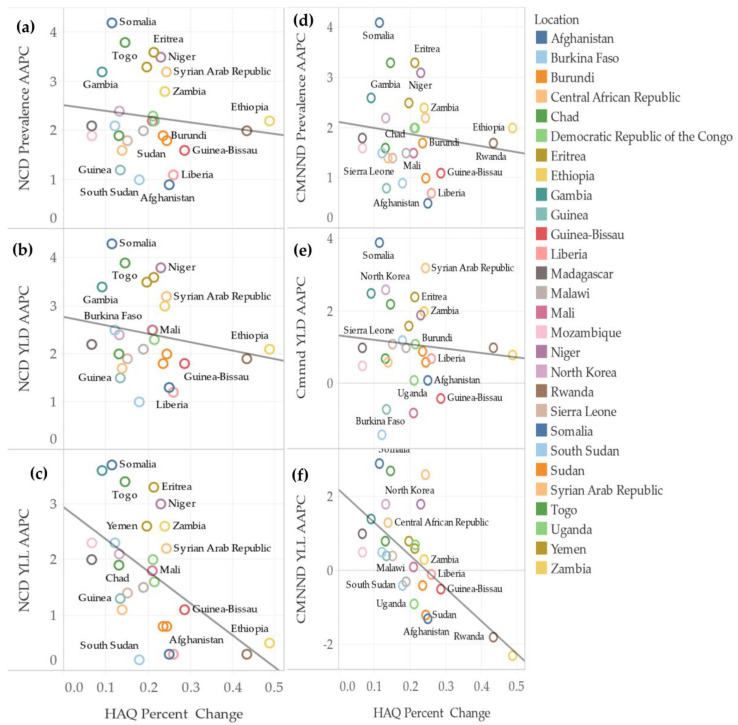
Correlations between Percentage Change in HAQ and CMNND Prevalence, YLD, and YLL AAPCs. HAQ percentage change = (HAQ in 1990/HAQ in 2019)/HAQ in 1990. All AAPCs represent the average annual percentage change in the number values of indicators by country for all years 1990–2019. Figure trend lines: (**a**) Rsquared = 0.02, *p*-value *=* 0.52; (**b**) Rsquared = 0.04, *p*-value *=* 0.33; (**c**) Rsquared = 0.27, *p*-value *=* 0.005; (**d**) Rsquared = 0.02, *p*-value *=* 0.49; (**e**) Rsquared = 0.01, *p*-value *=* 0.63; (**f**) Rsquared = 0.42, *p*-value *=* 0.0001.

The increase in HAQ was found to be associated with high AAPCs for ULYs among countries (Figure 2). On the other hand, AAPCs for YLDs and prevalence for both NCDs and CMNNDs showed no correlation with the percentage change in HAQ, but AAPCs for YLLs of both NCDs and CMNNDs had a moderately positive correlation with HAQ percentage change (Figure 3).

## 4. Discussion

This study explored the results of the GBD 2019 study and highlighted the increasing life expectancy and HALE among LICs. We also investigated the trend of HAQ among the countries and how this has correlated with healthcare needs, including YLD, prevalence and YLL in these countries.

LICs are perceived to have relatively younger populations, although they are home to 9 percent of the world’s population [24]. With projections of larger populations in the future, it is necessary to understand the current health issues facing elder people in these countries to plan for the future when these masses of young people turn old. Our findings emphasize the increasing number of prevalent cases, years of life lost, and years lived with disability in LICs for both CMNNDs and NCDs among the elderly. In his article, Boutayeb (2010) found that all over the world, both LICs and HICs were facing a growing “double burden” of CMNNDs and NCDs. He also found a highly increasing trend of NCDs in these populations, especially cardiovascular diseases (CVD) [25]. Our findings reaffirm this by showing a growing trend in both disease classifications. However, as YLDs and YLLs attributable to CMMNDs are reducing in some countries and having marginal increases in others, NCD measures, on the other hand, are increasing in all countries and at a higher rate than CMNNDs among elderly people. 

Previous publications have indicated the difference between CMNNDs and NCDs, ideally confirming the fact that while most CMMNDs are treatable and curable, NCDs pose a bigger challenge since, as much as they are treatable, many are not curable [26]. A GBD risk factor study found that minimal improvements have been made in reducing exposure to behavioral risks, such as secondhand smoke, alcohol use and dietary risks, among others. Moreover, metabolic risks are increasing, including high body mass index, high fasting plasma glucose, and high systolic blood pressure in low social development index countries [16]. NCDs are harder to prevent and control by governments than CMNNDs, and with increasing age comes an increased risk of suffering these diseases, meaning that the constant exposure of older people is bound to cause an increasing burden of disease [26].

There have been increases in life expectancy since 1990 among all countries, and this correlates with a fall in YLLs as there have been successes in the minimization of the effect of acute diseases and an increase in survival rates for people past the age of 65 [24]. At the same time, there has been an increase in HALE in all countries, which has also been correlated with higher mean years of schooling, a higher total fertility rate, and achieving high levels of health-related millennium goals among LICs and lower middle-income countries (LMICs) [27]. However, as people grow older, there have been concerns about the fact that the proportion of their disability and/or dependency-free life continues shrinking, suggesting expanding morbidity, a scenario undesirable and dubbed a “failure of success” in previous studies [13,28,29].

This means that due to the positive AAPCs in all NCD-related health care needs, including prevalence, YLDs and YLLs, which are concurrent with an increasing life expectancy and HALE, it is inevitable to have increasing numbers in ULYs, which have managed to continuously rise even as these countries have succeeded in lengthening the life. The expansion of morbidity has, however, been documented to exert higher demands on healthcare systems [13,30]. Moreover, it is a notable fact that the proportion the ULYs have on life expectancy has not changed that much throughout all LICs, indicating that the proportion of life spent in ill health has continued to stay the same even with achievements related to longer and healthier lives.

There is an increase in the HAQ index of all the countries studied, with high percentage increases for Ethiopia and Rwanda, but the index is still low among the LICs, with all of them being below the 50-point mark of a 100-point index in 2019. Yet, CAR posted a 17.16 score, which is the lowest. Since the index is an indicator of healthcare quality, provision and access, these results show that elders, especially post-working adults, do not have adequate healthcare in LICs. A study by GBD collaborators in 2015 indicated that indeed low-SDI countries have low HAQ Index scores, which is a consistent finding with our study [19]. 

The low HAQ for elders in countries with an increasing number of unhealthy years yet with increasing life expectancy and HALE raises a need for policy and academic insights to improve healthcare systems to meet the changing demographic landscape. 

To achieve an age-friendly healthcare system, improving healthcare access and quality for elders in these countries is essential. This will ensure more care and a possible reduction in morbidity. 

This study has some notable strengths. These include the addition of evidence on the scarcely documented issue of the increasing burden of NCDs among elders in LICs. It also covers a 30-year trend, indicating average annual percentage changes in indicators of the burden of disease for all LICs. This study also documents trends in life expectancy and HALE and computes estimations of ULYs. Providing a basis for further studies in the direction of aging and health in LICs.

This study also has limitations, the major of which is the use of data from the GBD 2019 study, but the study’s limitations have been critically documented [31]. The critics indicate a potential variance in the data quality, mostly among LICs, and our focus on older people in these countries makes it a more outstanding limitation as scarce data on the health of this population is available. HAQ variations are captured for only 1990 and 2019, yet there could be variations uncaptured during the 30 years studied.

## 5. Conclusions

As we acknowledge the success of health interventions in LICs in yielding increased life expectancy and HALE at birth, the health needs of elders are increasing, yet health systems demonstrate low scores when measured for access and quality towards these elders. LICs need to evolve as they eradicate CMNNDs among populations to be able to handle the worryingly increasing needs of NCD-laden elders in their populations. There is also a need to investigate further which components of health systems are ideal for increasing HAQ in LICs.

## Figures and Tables

**Table 1:** The Trend of Years Lived with Disability, Prevalence and Years of Life Lost due to Communicable, Malnutrition, Maternal, and Newborn Diseases between 1990 and 2019 for ages 64–75.

Communicable, Malnutrition, Maternal and Newborn Diseases							
Location	Year	Years Lived with Disability		Prevalence		Years of Life Lost	
		Number	AAPC	Number	AAPC	Number	AAPC
Afghanistan	1990	12,347.73 (7253.39–19,497.04)	0.1 (0.1–0.2)	317,971.79 (294,484.14–340,053.95)	0.5 (0.4–0.6)	60,147.12 (44,432.22–79,293.32)	−1.3 (−1.5–1.1)
	2019	12,734.99 (7962.69–19,091.75)		364,889.44 (332,189.33–399,138.78)		41,268.99 (30,826.5–51,631.84)	
Burkina Faso	1990	12,379.66 (8719.11–16,848.82)	−1.4 (−1.7–1.1)	226,774.17 (221,134.33–235,044.56)	1.5 (1.4–1.6)	122,232.08 (102,074.86–145,974.81)	0.5 (0.1–0.9)
	2019	7993.78 (5705.16–10,695.89)		350,667.92 (327,873.95–371,015.28)		138,954.42 (108,492.24–175,773.3)	
Burundi	1990	4207.67 (3027.98–5783.62)	0.9 (0.8–1)	112,439.98 (109,562.58–114,796.44)	1.7 (1.5–1.9)	71,742.12 (58,111.5–87,632.26)	−0.4 (−0.7–0.1)
	2019	5451.73 (3978.58–7306.05)		183,685.89 (176,850.32–189,330.78)		63,875.54 (50,037.24–80,608.83)	
Central African Republic	1990	3355.33 (2409.22–4393.83)	0.6 (0.4–0.7)	55,480.77 (54,223.8–57,029.37)	1.4 (1.4–1.5)	34,407.3 (28,066.91–41,452.4)	1.3 (1.2–1.5)
	2019	3959.78 (2919.95–5225.41)		84,422.26 (82,236.79–86,578.01)		49,770.57 (38,701.97–62,283.85)	
Chad	1990	5418.63 (3913.29–7164.26)	0.7 (0.5–1)	140,623.55 (135,625.57–145,503.27)	1.6 (1–2.1)	69,779.01 (56,673.4–82,888.22)	0.8 (0.5–1)
	2019	6671.36 (4817.57–8868.9)		219,547.98 (208,001.24–230,111.65)		86,348.14 (69,930.45–109,284.99)	
Democratic Republic of the Congo	1990	48,664.43 (34,603.4–64,518.59)	1.1 (1.1–1.2)	821,944.77 (812,358.29–831,096.43)	2 (2–2.1)	308,974.84 (247,522.54–378,554.12)	0.7 (0.5–1)
	2019	68,063.76 (48,938.65–89,031.82)		1,477,033.64 (1,444,898.32–1,504,401.1)		389,871.41 (304,097.65–495,335.14)	
Eritrea	1990	1185.8 (826.86–1593)	2.4 (2.2–2.5)	36,065.72 (34,893.83–37,058.11)	3.3 (3–3.5)	29,212.01 (21,023.48–36,625.16)	0.6 (0.5–0.8)
	2019	2340.17 (1645.95–3186.67)		90,778.57 (85,571.48–95,767.19)		35,112.67 (26,071.92–46,112.28)	
Ethiopia	1990	37,862.98 (27,610.18–50,408.21)	0.8 (0.7–0.9)	966,824 (944,746.14–985,525.92)	2 (1.9–2.1)	711,309.82 (604,381.4–822,846.88)	−2.3 (−2.5–2.1)
	2019	47,806.94 (34,180.59–64,022.71)		1,700,538.14 (1,637,789.52–1,754,371.39)		359,723.85 (307,975.63–419,789.92)	
Gambia	1990	398.46 (286.5–536.5)	2.5 (2.4–2.7)	15,089.65 (14,270.09–15,991.56)	2.6 (2.4–2.8)	7636.73 (5820.32–9846.74)	1.4 (0–2.8)
	2019	815.91 (575.75–1087.34)		31,843.74 (29,623.52–34,304.55)		11,963 (9068.92–17,698.2)	
Guinea	1990	6360.34 (4565.7–8504.04)	−0.7 (−0.8–0.6)	175,574.08 (170,900.48–180,319.52)	0.8 (0.7–1)	73,251.79 (60,021.94–88,102.6)	0.4 (0.2–0.6)
	2019	5236.22 (3758.71–7098.81)		226,365.73 (215,665.59–236,332.52)		82,254.7 (65,053.9–102,939.47)	
Guinea-Bissau	1990	734.52 (533.12–972.2)	−0.4 (−0.5–0.3)	20,076.17 (19,447.53–21,087.39)	1.1 (1–1.3)	12,148.74 (9960.03–14,721.8)	–0.5 (−0.7–0.3)
	2019	646.57 (462.75–880.62)		27,721.97 (26,278.68–29,285.98)		10,526.19 (8332.99–13,367)	
Liberia	1990	3490.22 (2483.69–4679.53)	0.7 (0.2–1.2)	63,588.32 (61,411.66–66,795.9)	0.7 (0.5–1)	27,024.4 (22,564.24–32,570.95)	−0.1 (−0.6–0.5)
	2019	4287.32 (3008.33–5871.36)		82,362.04 (79,616.34–85,705.7)		25,892.99 (18,740.7–35,475.17)	
Madagascar	1990	7105.1 (5089.18–9614.38)	1 (0.8–1.2)	235,081.12 (226,468.2–245,582.16)	1.8 (1.7–1.9)	77,840.92 (66,222.71–92,048.81)	1 (0.8–1.3)
	2019	9602.98 (6772.45–12,926.58)		393,359.45 (374,706.08–410,337.1)		103,310.53 (79,482.91–135,112.31)	
Malawi	1990	7572.69 (5488.74–9997.81)	1 (0.8–1.2)	197,377.36 (190,075.63–209,470.03)	1.5 (1.4–1.6)	104,903.52 (89,364.64–122,273.73)	−0.3 (−0.5–0.1)
	2019	10,076.61 (7119.53–13,462.33)		305,914.48 (292,954.12–318,265.43)		96,887 (81,513.92–114,847.25)	
Mali	1990	8905.85 (6400.62–11,860.53)	−0.8 (−1–0.7)	214,393.7 (208,595.31–221,472)	1.5 (1.5–1.6)	98,716.43 (83,252.18–117,849.81)	0.1 (−0.1–0.3)
	2019	7102.45 (5057.87–9613.65)		336,653.96 (316,750.47–357,369.01)		102,046.61 (79,054.37–136,006.59)	
Mozambique	1990	13,140.52 (9498.9–17,892.47)	0.5 (0.3–0.7)	301,192.19 (294,864.19–308,651.43)	1.6 (1.5–1.7)	161,900.83 (134,338.46–191,386.68)	0.5 (0.2–0.8)
	2019	14,970.53 (10,532.73–20,173.72)		474,792.01 (456,973.26–490,883.71)		187,313.89 (151,216.54–233,019.44)	
Niger	1990	4398.56 (3085.98–5915.81)	1.9 (1.4–2.4)	127,151.73 (121,233.61–135,179.4)	3.1 (2.2–4)	65,819.64 (52,884.14–80,461.25)	1.8 (1.4–2.2)
	2019	7373.56 (5186.38–9966.24)		307,072.13 (291,594.1–321,993.17)		111,928.98 (84,074.32–147,415.51)	
North Korea	1990	6507.57 (4567.38–8884.79)	2.6 (2.4–2.8)	554,364.01 (507,709.28–595,057.54)	2.2 (2.1–2.4)	28,012.52 (22,531.39–34,803.53)	1.8 (−8.1–12.7)
	2019	13,741.02 (9732.46–19,046.32)		1,065,269.83 (966,416.65–1,146,598.85)		42,497.22 (33,197.46–53,659.05)	
Rwanda	1990	4053.66 (2894.63–5521.01)	1 (0.1–1.9)	144,341.83 (140,685.93–147,250.81)	1.7 (0.9–2.5)	81,818.53 (64,686.43–100,580.85)	−1.8 (−2.2–1.3)
	2019	5710.5 (4067.5–7723.82)		245,234.17 (233,647.45–256,564.26)		48,736.95 (39,529.5–60,032.1)	
Sierra Leone	1990	3850.23 (2785.55–5062.17)	1.1 (0.9–1.3)	96,746.89 (92,841.99–101,957.86)	1.4 (1.2–1.5)	51,052.13 (40,653.22–62,571.82)	0.4 (0.3–0.6)
	2019	5332.27 (3708.77–7373.42)		144,267.09 (137,534.91–152,416.27)		57,767.41 (42,101.96–76,990.45)	
Somalia	1990	3111.32 (2156.27–4273.46)	3.9 (3.9–4)	92,648.78 (90,464.77–94,574.35)	4.1 (4.1–4.2)	60,416.62 (48,044.87–75,385.22)	2.9 (2.5–3.3)
	2019	9542.08 (6676.76–13,138.48)		296,987.07 (288,447.66–306,067.2)		137,131.29 (103,959.34–177,633.43)	
South Sudan	1990	7566.86 (5376.37–10,123.21)	1.2 (1.1–1.3)	118,158.75 (115,796.55–120,270.71)	0.9 (0.8–1)	47,794.28 (38,059.36–59,241.87)	−0.4 (−0.5–0.2)
	2019	10,657.41 (7576.47–14,310.1)		154,351.27 (150,268.17–157,823.63)		43,235.99 (32,022.71–55,240.49)	
Sudan	1990	7791.63 (5521.45–10,849.52)	0.6 (0.6–0.7)	390,514.11 (360,346.69–424,279.47)	1 (1–1.1)	49,344.82 (28,897.11–69,535.02)	−1.2 (−1.4–0.9)
	2019	9415.34 (6590.12–12,770.55)		523,908.14 (470,631.71–583,239.11)		35,497.2 (26,167.4–46,772.03)	
Syrian Arab Republic	1990	1909.04 (1292.59–2741.05)	3.2 (3.1–3.4)	162,961.97 (145,575.8–179,184.71)	2.2 (2.1–2.3)	4972.61 (3552.57–6467.37)	2.6 (2.1–3.1)
	2019	4782.62 (3364.31–6574.66)		309,094.31 (278,459.22–345,327.48)		10,797.48 (7685.54–14,397.92)	
Togo	1990	1860.36 (1361.13–2467.49)	2.2 (2–2.4)	54,362.7 (52,506.71–56,142.97)	3.3 (3–3.6)	21,958.83 (18,285.16–26,704.03)	2.7 (2.5–2.9)
	2019	3453.18 (2495.84–4620.47)		138,738.15 (132,351.96–145,651.81)		47,862.22 (36,835.35–63,053.23)	
Uganda	1990	15,329.72 (11,154.77–20,058.19)	0.1 (−0.1–0.3)	327,027.48 (323,946.28–330,201.62)	2 (1.9–2.1)	166,509.89 (138,656.52–201,060.18)	−0.9 (−1.3–0.4)
	2019	15,654.98 (11,230.9–20,772.78)		581,665.44 (566,081.62–593,964.53)		130,492.87 (107,242.89–157,076.69)	
Yemen	1990	6332.3 (4173.31–9328.21)	1.6 (1.5–1.8)	211,757.82 (200,906.44–220,876.55)	2.5 (2.4–2.6)	27,030.26 (16,970.61–41,697.91)	0.8 (0.5–1.2)
	2019	10,124.04 (6832.58–14,632.21)		440,467.65 (401,405.84–479,955.75)		35,711.72 (22,059.51–63,177.11)	
Zambia	1990	4610.46 (3333.04–6125.41)	2 (1.9–2.2)	126,703.8 (123,577.17–130,186.22)	2.4 (2.4–2.5)	64,028.4 (51,971.25–75,306.55)	0.3 (0–0.5)
	2019	8247.51(5962.88–11,106.88)		256,291.48(246,022.14–265,856.46)		69,637.2(56,914.18–85,997.48)	

Value (Lower UI-Upper UI), AAPC; Average annual percentage change.

**Table 2:** The Trend of Years Lived with Disability, Prevalence and Years of Life Lost due to Non-Communicable Diseases between 1990 and 2019 for ages 64–75.

Non-Communicable Diseases							
Location	Year	Years Lived with Disability		Prevalence		Years of Life Lost	
		Number	AAPC	Number	AAPC	Number	AAPC
Afghanistan	1990	74,520.56 (56,105.14–95,556.59)	1.3 (1.1–1.4)	391,648.55 (391,449.11–391,815.74)	0.9 (0.8–1.1)	431,724.28 (363,133.76–504,800.31)	0.3 (0.2–0.4)
	2019	106,933.52 (79,792.37–136,530.93)		510,237.19 (510,051.75–510,396.62)		468,700.74 (379,901.91–539,617.62)	
Burkina Faso	1990	38,346.09 (28,980.99–49,081.31)	2.5 (2.3–2.8)	236,711.12 (236,578.16–236,839.98)	2.1 (2–2.3)	127,942.41 (104,704.6–148,929.18)	2.3 (2.2–2.5)
	2019	78,362.75 (59,051.76–100,396.49)		435,127.11 (434,923.74–435,300.1)		243,778.74 (202,101–285,653.42)	
Burundi	1990	20,842.74 (15,908.03–26,565.78)	1.8 (1.7–1.9)	119,880.5 (119,772.27–119,977.49)	1.9 (1.9–2)	102,262.26 (86,043.88–119,936.65)	0.8 (0.6–1.1)
	2019	35,118.06 (26,579.52–44,862.01)		209,848.59 (209,619.56–210,059.47)		128,959.69 (106,480.3–160,163.19)	
Central African Republic	1990	10,054.11 (7687.13–12,775.15)	1.7 (1.7–1.8)	57,537 (57,504.02–57,567.33)	1.6 (1.5–1.7)	55,916.74 (48,193.16–63,814.94)	1.1 (0.9–1.3)
	2019	16,608.61 (12,617.28–21,076.53)		91,266.6 (91,210.52–91,312.56)		76,899.96 (61,099.81–94,452.2)	
Chad	1990	27,588.17 (20,847.58–35,051.7)	2 (1.5–2.5)	158,324.81 (158,215.7–158,422.61)	1.9 (1.4–2.3)	94,790.07 (81,228.66–108,803.97)	1.9 (1.5–2.3)
	2019	49,608.34 (37,426.3–63,512.95)		270,274.35 (270,095.11–270,424.6)		162,148.74 (135,547.34–191,859)	
Democratic Republic of the Congo	1990	145,208.44 (110,047.83–185,011.4)	2.3 (2.3–2.4)	851,005.68 (850,410.99–851,501.8)	2.2 (2.1–2.2)	642,374.13 (554,290.63–742,393.28)	1.6 (1.4–1.7)
	2019	283,696.87 (214,153.3–361,227.49)		1,593,243.24 (1,592,356.26–1,594,084.51)		1,004,552.8 (836,353.25–1,207,100.23)	
Eritrea	1990	7128.98 (5445.83–9066.23)	3.6 (3.4–3.9)	41,214.45 (41,178.15–41,247.72)	3.6 (3.3–3.8)	29,846.55 (23,091.97–38,079.23)	3.3 (2.7–3.8)
	2019	19,896.03 (15,100.13–25,485.07)		113,421.24 (113,319.19–113,504.85)		75,926.88 (60,817.15–91,849.33)	
Ethiopia	1990	187,821.96 (142,553.15–238,839.06)	2.1 (2–2.3)	1,048,773.24 (1,047,883.27–1,049,601.18)	2.2 (2.1–2.3)	876,575.53 (762,360.5–983,993.05)	0.5 (0.3–0.6)
	2019	346,907.45 (261,674.9–445,017.94)		1,982,998.56 (1,981,292.14–1,984,593.08)		1,000,815.36 (890,291.35–1,103,143.67)	
Gambia	1990	3156.85 (2389.41–4025.48)	3.4 (3.3–3.6)	17,809.1 (17,797.38–17,819.24)	3.2 (3.1–3.3)	9795.42 (7663.87–12,164.84)	3.6 (2.6–4.5)
	2019	8415.98 (6332.4–10,726.53)		44,590.51 (44,563.88–44,611.87)		26,179.98 (20,608.75–31,269.1)	
Guinea	1990	32,103.99 (24,307.08–41,026.82)	1.5 (1.4–1.7)	190,567.42 (190,446.99–190,678.36)	1.2 (1.1–1.4)	119,789.04 (103,296.23–135,963.27)	1.3 (1.2–1.4)
	2019	50,067.58 (37,699.86–63,985.85)		274,302.54 (274,144.96–274,435.23)		172,890.1 (142,254.64–207,033.25)	
Guinea-Bissau	1990	3751.29 (2851.9–4759.98)	1.8 (1.8–1.9)	21,340 (21,326.08–21,351.84)	1.6 (1.6–1.6)	18,615.2 (15,509.89–21,719.53)	1.1 (1–1.1)
	2019	6336.87 (4780.93–8066.19)		33,792.3 (33,772.54–33,808.73)		25,022.5 (20,535.02–29,794.5)	
Liberia	1990	11,591.79 (8839.47–14,726.68)	1.2 (1–1.4)	66,713.4 (66,672.83–66,748.91)	1.1 (0.8–1.3)	40,462.18 (34,413.49–46,656.83)	0.3 (−0.3–0.9)
	2019	16,535.7 (12,483.38–21,049.38)		91,216.54 (91,169.04–91,258.56)		44,960.14 (35,062.49–56,765.49)	
Madagascar	1990	46,070.58 (35,014.14–58,507.62)	2.2 (2.1–2.3)	258,875.89 (258,681.2–259,043.74)	2.1 (2–2.2)	176,317.54 (158,018.12–195,720.98)	2 (1.6–2.3)
	2019	86,542.82 (66,216.38–109,889.8)		474,706.06 (474,363.72–475,012.89)		313,468.84 (253,699.07–388,304.55)	
Malawi	1990	37,338.45 (28,266.1–47,275.39)	2.1 (2–2.2)	211,150.98 (211,002–211,292.63)	2 (1.9–2.1)	136,923.81 (118,863.36–153,764.99)	1.5 (1.4–1.7)
	2019	67,876.29 (51,516.69–86,680.93)		375,587.03 (375,350.84–375,803.65)		215,661.38 (184,621.55–249,123.56)	
Mali	1990	37,580.28 (28,375.14–47,692.69)	2.5 (2.4–2.5)	221,151.71 (220,992.67–221,295.83)	2.2 (2.2–2.3)	141,841.47 (122,444.77–160,186.27)	1.8 (1.6–1.9)
	2019	76,842.66 (58,056.04–98,092.69)		421,934.69 (421,683.62–422,157.04)		234,945.11 (195,938.5–286,808.94)	
Mozambique	1990	52,855.72 (40,216.52–67,475.37)	2.2 (2.1–2.3)	308,480.97 (308,217.77–308,718.14)	1.9 (1.9–2)	184,105.99 (155,814.57–214,622.08)	2.3 (2.2–2.4)
	2019	99,155.99 (74,647.45–126,058.33)		538,806.14 (538,422.23–539,170.67)		357,416.19 (297,691.1–429,024.54)	
Niger	1990	22,588.83 (17,057.39–28,710.95)	3.8 (2.9–4.6)	138,562.12 (138,474.78–138,633.57)	3.5 (2.6–4.4)	85,886.12 (72,215.89–99,420.07)	3 (2.3–3.7)
	2019	65,660.81 (49,516.73–83,902.89)		376,371.95 (376,174.45–376,533.44)		199,603.26 (160,316.9–241,892.48)	
North Korea	1990	152,435.78 (116,320.54–191,299.92)	2.4 (2.3–2.5)	772,192.37 (771,298–772,985.45)	2.4 (2.2–2.6)	550,814.81 (466,766.9–639,441.08)	2.1 (1.9–2.2)
	2019	307,089.42 (234,289.53–385,144.82)		1,548,852.5 (1,547,190.83–1,550,342.04)		999,945.71 (896,116.44–1,100,308.76)	
Rwanda	1990	28,322.53 (21,488.39–35,880.94)	1.9 (1.5–2.4)	155,737.21 (155,579.27–155,884.74)	2 (1.6–2.5)	140,207.75 (120,958.44–160,152.43)	0.3 (−0.7–1.3)
	2019	51,665.29 (39,114.75–66,202.9)		295,160.86 (294,837.24–295,448.29)		161,301.51 (141,517.67–187,885.68)	
Sierra Leone	1990	17,090.29 (13,000.17–21,796.21)	1.9 (1.8–2)	101,836.62 (101,770.75–101,890.27)	1.8 (1.7–1.9)	60,522.89 (49,600.58–71,902.81)	1.4 (1–1.9)
	2019	29,585.9 (22,423.68–37,975.42)		170,767.87 (170,660.03–170,861.46)		90,789.6 (70,997.71–114,098.28)	
Somalia	1990	17,203.1 (13,106.99–21,954.16)	4.3 (4.3–4.4)	100,667.96 (100,583.91–100,747.89)	4.2 (4.2–4.3)	81,459.33 (65,099.42–99,008.95)	3.7 (3.6–3.8)
	2019	58,493.42 (44,511.47–75,094.27)		334,342.11 (334,061.26–334,592.64)		234,479.41 (186,492.43–294,079.96)	
South Sudan	1990	22,329.31 (17,095.84–28,156.67)	1 (0.8–1.1)	126,551.54 (126,436.58–126,653.37)	1 (0.8–1.1)	81,844.8 (66,475.37–96,999.14)	0.2 (0.1–0.4)
	2019	29,828.99 (22,697.72–37,887.66)		169,118 (168,979.56–169,245.96)		88,567.39 (68,864.69–109,752.58)	
Sudan	1990	98,042.53 (73,211.46–125,181.66)	2 (1.9–2)	512,013.25 (511,710.5–512,269.35)	1.8 (1.8–1.8)	437,874.04 (388,288.81–488,005.38)	0.8 (0.8–0.9)
	2019	171,763.92 (128,217.39–219,354.22)		859,907.52 (859,504.93–860,273.16)		557,511.15 (478,198.6–673,077.7)	
Syrian Arab Republic	1990	49,242.25 (36,889.74–63,063.06)	3.2 (3.1–3.3)	253,577.19 (253,458.94–253,680.29)	3.2 (3.1–3.2)	181,428.41 (149,983.52–214,493.56)	2.2 (1.9–2.5)
	2019	123,416.65 (91,466.73–159,442.55)		633,482.31 (633,237.15–633,677.06)		354,047.72 (271,174.42–460,684.76)	
Togo	1990	10,411.84 (7905.82–13,244.26)	3.9 (3.6–4.3)	60,307.96 (60,267.66–60,343.16)	3.8 (3.7–3.9)	37,269.69 (32,258.9–43,057.67)	3.4 (2.8–4.1)
	2019	31,450.54 (23,807.86–40,266.26)		173,145.02 (173,043.81–173,226.19)		95,062.44 (78,015.99–115,317.46)	
Uganda	1990	58,512.5 (44,278.15–74,511.86)	2.5 (2.3–2.6)	335,148.07 (334,851.87–335,457.84)	2.3 (2.2–2.5)	208,065.51 (175,816.77–237,115.38)	2 (1.8–2.1)
	2019	117,926.88 (88,714.73–150,040.67)		647,607.3 (647,080.84–648,086.44)		371,750.02 (317,452.3–418,787.87)	
Yemen	1990	46,176.83 (34,600.61–58,852.13)	3.5 (3.4–3.6)	252,099.52 (251,965.3–252,213.86)	3.3 (3.3–3.4)	219,839.69 (185,109.88–261,101.71)	2.6 (2.5–2.7)
	2019	124,923.26 (92,991.2–160,323.72)		652,613.43 (652,312.84–652,884.11)		463,352.78 (394,465.75–569,843.65)	
Zambia	1990	22,625.32 (17,132–28,802.94)	3 (2.9–3)	134,423.23 (134,322.23–134,518.75)	2.8 (2.7–2.8)	98,039.2 (85,620.77–110,870.16)	2.6 (2.5–2.6)
	2019	52,979.9(40,102.06–67,736.48)		299,363.9(299,162.63–299,535.54)		204,653.59(172,114.28–241,168.82)	

Value (Lower UI-Upper UI), AAPC; Average annual percentage change.

**Table 3:** Unhealthy life years (ULYs) and their percentage of life expectancy.

Location	UL Ys		Proportion of ULYs	
	1990	2019	1990	2019
Afghanistan	8.20	9.22	15%	15%
Burkina Faso	6.61	7.51	13%	12%
Burundi	5.82	8.33	12%	13%
Central African Republic	6.42	6.64	13%	13%
Chad	6.68	7.53	12%	12%
Democratic Republic of the Congo	7.66	8.71	14%	13%
Eritrea	6.17	7.94	13%	12%
Ethiopia	5.64	8.62	12%	13%
Gambia	7.47	8.43	12%	13%
Guinea	6.33	7.37	12%	12%
Guinea-Bissau	5.75	7.34	12%	12%
Liberia	7.12	9.22	14%	14%
Madagascar	7.07	7.88	13%	12%
Malawi	6.27	8.13	13%	13%
Mali	6.30	7.57	13%	12%
Mozambique	6.97	7.58	14%	13%
Niger	5.67	7.45	12%	12%
North Korea	7.51	8.18	11%	11%
Rwanda	6.02	8.82	12%	13%
Sierra Leone	6.51	7.72	13%	12%
Somalia	5.83	7.01	12%	12%
South Sudan	7.52	9.14	14%	14%
Sudan	7.22	9.07	12%	13%
Syrian Arab Republic	8.39	9.99	12%	14%
Togo	7.24	8.02	12%	12%
Uganda	6.44	8.47	13%	13%
Yemen	7.64	9.16	13%	14%
Zambia	6.52	8.10	13%	13%

**Table 4:** HAQ Trend between 1990 and 2019 (65–74 Years).

Country	1990	2019	Difference	%Change
Afghanistan	22.27 (18.08–27.03)	29.64 (25.53–35.21)	7.36793768	25%
Burkina Faso	25.8 (20.75–30.83)	29.33 (25.02–33.88)	3.5294123	12%
Burundi	18.72 (14.12–23.61)	24.45 (20.09–28.82)	5.7233613	23%
Central African Republic	14.81 (10.72–19.87)	17.16 (12.34–22.42)	2.35718068	14%
Chad	23.04 (18.67–27.65)	26.48 (22.09–30.87)	3.43930043	13%
Democratic Republic of the Congo	21.46 (17.15–25.92)	27.29 (22.73–32.07)	5.8268967	21%
Eritrea	18.22 (13.41–24.68)	23.13 (18.75–28.15)	4.91004852	21%
Ethiopia	15.03 (11.11–19.65)	29.22 (24.93–34.1)	14.18889766	49%
Gambia	28.82 (23.56–34.33)	31.67 (27.33–37.2)	2.85319467	9%
Guinea	25.25 (20.85–29.67)	29.12 (24.82–33.86)	3.87481994	13%
Guinea-Bissau	16.58 (11.8–22)	23.18 (19.19–27.16)	6.60557121	28%
Liberia	26.47 (21.93–31.36)	35.7 (30.46–40.98)	9.22809427	26%
Madagascar	26.77 (22.54–31.57)	28.65 (23.31–34.06)	1.87901438	7%
Malawi	22.82 (19.21–27.27)	28.1 (24.72–31.87)	5.27600946	19%
Mali	25.05 (20.11–30.23)	31.65 (26.64–36.98)	6.60425469	21%
Mozambique	21.11 (16.93–25.83)	22.61 (18.89–26.65)	1.49286003	7%
Niger	21.44 (16.77–26.77)	27.8 (22.66–33.67)	6.3612372	23%
North Korea	39.46 (35.17–43.86)	45.39 (41.59–49.13)	5.93822538	13%
Rwanda	17.44 (13.78–21.62)	30.7 (27.07–34.25)	13.26094126	43%
Sierra Leone	27.4 (22.56–32.47)	32.23 (27.37–37.3)	4.83490059	15%
Somalia	17.42 (12.12–22.41)	19.65 (13.67–25.36)	2.22732602	11%
South Sudan	24.86 (20.24–30.07)	30.24 (24.47–36.55)	5.38086452	18%
Sudan	34.01 (29.65–38.69)	44.93 (39.58–49.33)	10.91835207	24%
Syrian Arab Republic	45.39 (40.63–50.51)	59.89 (54.63–65.01)	14.49606358	24%
Togo	26.73 (22.72–31.06)	31.24 (27.01–35.24)	4.50894049	14%
Uganda	24.41 (20.3–28.96)	30.91 (27.49–34.72)	6.49667434	21%
Yemen	30.92 (26.22–35.79)	38.44 (33.97–43.02)	7.52061641	20%
Zambia	22.18 (18.41–26.46)	29.12 (25.11–34.08)	6.93552929	24%

## Data Availability

The data that supports the findings and derived conclusions in this study are publicly available in the GBD Results Tool (http://ghdx.healthdata.org/gbd-results-tool (accessed on 2 February 2023)).
